# International law’s effects on health and its social determinants: protocol for a systematic review, meta-analysis, and meta-regression analysis

**DOI:** 10.1186/s13643-016-0238-0

**Published:** 2016-04-16

**Authors:** Steven J. Hoffman, Matthew Hughsam, Harkanwal Randhawa, Lathika Sritharan, Gordon Guyatt, John N. Lavis, John-Arne Røttingen

**Affiliations:** Global Strategy Lab, Faculty of Law, University of Ottawa, Fauteux Hall, 57 Louis Pasteur St, Ottawa, K1N 6N5 Ontario Canada; Bachelor of Health Sciences Program, McMaster University, Hamilton, Ontario Canada; Michael G. DeGroote School of Medicine, McMaster University, Hamilton, Ontario Canada; Department of Clinical Epidemiology & Biostatistics, McMaster University, Hamilton, Ontario Canada; McMaster Health Forum, McMaster University, Hamilton, Ontario Canada; Division of Infection Control and Environmental Health, Norwegian Institute of Public Health, Oslo, Norway

**Keywords:** International cooperation, Global health, Social determinants of health, Public policy, Jurisprudence, Treaties

## Abstract

**Background:**

In recent years, there have been numerous calls for global institutions to develop and enforce new international laws. International laws are, however, often blunt instruments with many uncertain benefits, costs, risks of harm, and trade-offs. Thus, they are probably not always appropriate solutions to global health challenges. Given these uncertainties and international law’s potential importance for improving global health, the paucity of synthesized evidence addressing whether international laws achieve their intended effects or whether they are superior in comparison to other approaches is problematic.

**Methods:**

Ten electronic bibliographic databases were searched using predefined search strategies, including MEDLINE, Global Health, CINAHL, Applied Social Sciences Index and Abstracts, Dissertations and Theses, International Bibliography of Social Sciences, International Political Science Abstracts, Social Sciences Abstracts, Social Sciences Citation Index, PAIS International, and Worldwide Political Science Abstracts. Two reviewers will independently screen titles and abstracts using predefined inclusion criteria. Pairs of reviewers will then independently screen the full-text of articles for inclusion using predefined inclusion criteria and then independently extract data and assess risk of bias for included studies. Where feasible, results will be pooled through subgroup analyses, meta-analyses, and meta-regression techniques.

**Discussion:**

The findings of this review will contribute to a better understanding of the expected benefits and possible harms of using international law to address different kinds of problems, thereby providing important evidence-informed guidance on when and how it can be effectively introduced and implemented by countries and global institutions.

**Systematic review registration:**

PROSPERO CRD42015019830

**Electronic supplementary material:**

The online version of this article (doi:10.1186/s13643-016-0238-0) contains supplementary material, which is available to authorized users.

## Background

In recent years, there have been numerous calls for global institutions to develop and enforce new international laws in the realm of health. These international laws, which regulate relations between and within countries, vary widely in their subject matter. Focus areas for these calls have included alcohol [1], antimicrobial resistance [2–10], chronic diseases [11], falsified/substandard medicines [12], health system corruption [13], impact evaluations [14], nutrition [15], obesity [16], research and development [17, 18], and global health broadly [19]. Various stakeholders, including individual experts and intergovernmental organizations, have prompted these calls for new international treaties. Commonly cited evidence to support these proposals are past “successful” global health laws, including the World Health Organization’s *Framework Convention on Tobacco Control *(2003) and the *International Health Regulations *(2005) [20].

Although few international laws have been adopted specifically to promote human health, many international laws have possible indirect effects on health, as they may impact the social determinants of health (i.e., the external conditions in which people live that may affect their health). Examples of social determinants of health include armed conflict, employment, empowerment, environment, finance, human rights, poverty, sanitation, social policies, trade, and water supply [21, 22]. One prominent example of such an international law is the World Trade Organization’s *Agreement on Trade-Related Aspects of Intellectual Property Rights *(TRIPS). This law serves as the de facto global regulating policy for all forms of intellectual property, including copyrights, patents, and trademarks. The rules set out by this agreement have major implications for the medical product industry and thereby influence global access to medicines [23, 24].

While many stakeholders have advocated for international laws that provide solutions to complex global health challenges [1–19], others have raised concerns about international law being a blunt instrument with many uncertain benefits, costs, risks of harm, and trade-offs [25–30]. Few calls for new laws fully consider their potentially coercive and potentially paternalistic nature or the direct costs associated with drafting, ratification, and enforcement, including numerous meetings, legal fees, and duplicative governance structures that must be supported by governments. Further, the indirect opportunity costs associated with the resources, energy, and rhetorical space that go into negotiating international laws and implementing them may draw attention away from other potentially more important initiatives [25–30].

Given these uncertainties associated with international law, and its ever-increasing popularity as a potential tool for global change, there is surprisingly little synthesized evidence on whether international laws achieve their intended results or whether they are superior to other available instruments for coordinating international actors. The current lack of synthesized evidence on the effectiveness of international law has posed a significant barrier to understanding the potential value, feasibility, and applicability of international law for global health and related challenges.

A scoping review by Hoffman and Røttingen (2015) attempted to delineate the effects of international law by qualitatively summarizing 90 quantitative impact evaluations of different international laws [28]. That review, which is the most comprehensive to date, found that the effects of international laws were mixed and dependent on the nature of the instrument, its intended outcome, and mediating factors. While some international laws may promote beneficial outcomes, this may not always be the case. This recent review did not follow a systematic search or analysis protocol and was unable to produce a comprehensive analysis or an authoritative list of considerations.

This planned systematic review will build on the approach and findings of that recent scoping review, applying subgroup analysis, meta-analysis, and meta-regression techniques to synthesize all available quantitative research evidence on the effects of international law on health and its social determinants. Specifically, this review aims to answer the following three questions:

1. What are the effects of international law on health behaviors and outcomes?
2. What are the effects of international law on the social determinants of health?
3. Upon what factors are effects of international law conditional?

For example, in the context of the *Framework Convention on Tobacco Control *(FCTC), which is a global health treaty providing an internationally coordinated response to combatting tobacco use [20], the review would aim to identify:

1. What are the effects of the FCTC on health behaviors and outcomes like tobacco consumption, lung cancer rates, and life expectancy?
2. What are the effects of the FCTC on social determinants of health like tobacco advertising bans, tobacco taxes, and smoke-free workplace policies?
3. Upon what factors are the effects of the FCTC conditional, such as a country’s development status, regulatory capacity, and government ownership of tobacco companies?

This systematic review will boost current understanding on whether and how countries and global institutions might employ international law as a population health intervention for addressing today’s pressing global health challenges.

## Methods/design

### Protocol and registration

We will conduct this systematic review, meta-analysis, and meta-regression adhering to the following protocol and will report any changes to the protocol that arise as we proceed. The methods and design of this systematic review are based on recommendations from the Reporting Items for Systematic Reviews and Meta-Analyses (PRISMA-P) statement [31]. See Additional file 1 for the PRISMA-P 2015 checklist. The protocol is registered with the PROSPERO international prospective register of systematic reviews (registration number CRD42015019830).

### Types of study designs

All quantitative impact evaluations that measure the effect of an international law on outcomes related to health or its social determinants will be included. These may include experiments (e.g., randomized controlled trials), quasi-experiments (e.g., interrupted time-series analyses), and observational designs (e.g., pooled time-series cross-sectional analyses, event histories, survival analyses).

### Types of participants

Given that international law only imposes obligations on states (with only a small number of exceptions such as crimes against humanity for which individuals can also be convicted), “participants” will include all current and former countries, formally recognized as states by the international community, which is best indicated by membership in the United Nations or a similar multilateral organization. We will interpret this definition broadly to include jurisdictions that are often viewed as states by some entities (e.g., Monaco, Palestine, Taiwan, Vatican City).

### Types of interventions

Any implementation of international law related to health or its social determinants between at least two states will be considered as an intervention. International law is commonly referred to as agreements, charters, conventions, declarations, exchange of notes, memorandums of understanding, modus vivendi, protocols, treaties, and at least 30 other names. It is defined as the “rules and principles of general application dealing with the conduct of states and of international organizations and with their relations inter se, as well as with some of their relations with persons” [32]. Randomized controlled interventions are not necessary for studies to meet eligibility for inclusion in this review. Examples of possible control interventions include, but are not limited to, the absence of an international law or a local state law.

### Type of outcomes

To be included, a study has to measure the effect of international law on an objective and quantifiable outcome related to health or its social determinants, which, according to the World Health Organization’s Commission on the Social Determinants of Health (2008), include armed conflict, employment, empowerment, environment, finance, human rights, poverty, sanitation, social policies, trade and water supply, and among other factors [22]. Outcomes will be broadly categorized as changes to *people *(e.g., health status), *places *(e.g., carbon emissions), *products *(e.g., availability of medicines), or *policies *(e.g., new regulations or taxes). As this is an exploratory review, we have not specified exact outcomes a priori and have no reason to believe that one outcome will be more important than another. We will base the outcomes upon what data is available in the set of included studies and treat all outcomes as primary outcomes. However, we will preferentially extract and report outcomes that are found to have more data.

### Search strategy

The search strategy for this review was developed in consultation with one health sciences librarian at McMaster University and one at the University of Toronto. The following ten electronic bibliographic databases were searched from inception to July 2014: MEDLINE, Global Health, Cumulative Index to Nursing and Allied Health Literature (CINAHL), Applied Social Sciences Index and Abstracts, International Bibliography of Social Sciences, International Political Science Abstracts, Social Sciences Abstracts, Social Sciences Citation Index, Public Affairs Information Service International (PAIS International), and Worldwide Political Science Abstracts. To find gray literature, we also conducted an additional search in ProQuest’s Dissertations and Theses electronic database. No language, geography, or date restrictions were applied to the searches.

The exact search conducted in MEDLINE is presented in Table 1. See Additional file 2 for a list of the exact searches conducted in all ten electronic bibliographic databases and the dissertations and theses database.

**Table 1:** Search terms used in Ovid MEDLINE

MEDLINE search
Citations yielded = 576
1. (law or laws or agreement* or treaty or treaties or convention* or accord or accords or covenant* or protocol* or charter or charters or regime* or cooperation* or legislation*).tw.
2. (international or global or multi?national or trans?national or foreign or multi?lateral).tw.
3. (quantitative or empirical or experiment or experiments or experimental or quasi-experiment or quasi-experiments or quasi-experimental or statistic* or time?series or cross?sectional or tscs or counterfactual or ANOVA or MANOVA or t-test or z-test or f-test or logistic or correlation or frequentist or Bayesian or maximum likelihood or least squares or parametric or covariance).tw.
4. (effect* or affect* or impact* or ratif* or difference or differences or compliance or comply or adher* or implement* or influenc* or impact* or chang* or measur* or constrain* or screen* or behavio?r or deter* or reduc* or increas* or decreas* or inflat* or vary or variation* or varie*).tw.
5. ((law or laws or agreement* or treaty or treaties or convention* or accord or accords or covenant* or protocol* or charter or charters or regime* or cooperation or legislation*) adj3 (international or global or multi?national or trans?national or foreign or multi?lateral)).tw.
6. 3 and 4 and 5

### Study selection

This systematic review will include any published or unpublished study that aims to quantitatively measure the impact of an international law’s effect on any health-related behavior or outcome, including social determinants of health [22]. Studies will be included if the answer is “yes” to all of the following questions:

1. Is the study a quantitative impact evaluation?
2. Are the participants current or former countries?
3. Is there an international law being discussed?
4. Are the effects of an international law being measured? (i.e., the effects on health and its social determinants)

**Fig. 1:**
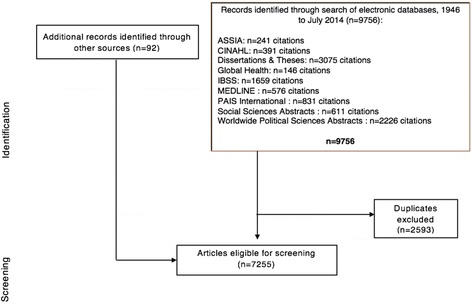
PRISMA flow diagram (in process). Legend:*ASSIA*Applied Social Sciences Index and Abstracts,*CINAHL*Cumulative Index to Nursing and Allied Health Literature,*IBSS*International Bibliography of Social Sciences,*PAIS International*Public Affairs Information Service International

Two reviewers will independently evaluate titles and abstracts to determine whether or not each article might meet eligibility criteria. The initial title and abstract screening process will make use of the aforementioned four criteria to assess whether articles will be assessed for inclusion at the full-text screening stage. For all studies included after title and abstract screening, four pairs of reviewers will independently screen the full-text of articles for inclusion, applying the same four criteria. A codebook containing examples for all four inclusion criteria will be developed to help guide reviewers through the screening process. Prior to full-text screening, multiple rounds of calibration exercises will be conducted to ensure that there is near-perfect consistency among reviewers. Each round of calibration will be followed by a meeting with all reviewers to resolve any discrepancies. All disagreements that arise during full-text screening will be documented and resolved through discussion and consensus; if consensus cannot be reached, the principal investigator (SJH) will settle disagreements (Fig. 1).

Additional records found in the recent scoping review [28] will go straight to full-text screening, bypassing title and abstract screening, because they are very likely eligible for inclusion.

### Data management and extraction

Search results from all databases will be aggregated in the reference management software EndNote, which will also be used to remove duplicate citations.

A statistician has been contracted to aid in developing the data abstraction form so as to ensure comprehensive extraction methods that will allow for pooling and analysis of results. Additionally, calibration exercises will be conducted to ensure that there is consistency between reviewers. These exercises will serve to pretest the form before it is used to extract data from eligible studies. An online data abstraction program (Distiller SR) will be used to maximize efficiency—it will be customized for this task using the criteria of a standardized form. Two pairs of reviewers will independently extract data from each eligible study. Data extracted will include general study information, methodology, intervention details, and outcome data. Any disagreements will be documented and resolved through discussion and consensus; when consensus cannot be reached, the principal investigator will settle disagreements.

### Risk of bias (quality) assessment

Studies that are consistent with the inclusion criteria will be assessed for bias using Cochrane’s ROBINS-I tool for non-randomized studies of interventions [33]. We do not anticipate the inclusion of any relevant randomized trials given the nature of the intervention (i.e., randomizing countries to receive an international law would not be politically feasible). Note that although the kinds of biases are broadly equivalent for randomized and non-randomized studies—namely selection, confounding, group equivalence, spill-overs, and reporting biases—there are important differences in their operationalization. Reviewers will resolve disagreements by discussion and through consultation with the principal investigator if necessary.

In presenting the evidence, the Grading of Recommendations Assessment, Development and Evaluation (GRADE) system of rating the quality of evidence will be used, as recommended by the Cochrane Collaboration [34].

### Strategy for data synthesis

Three types of analyses are currently planned, resulting in at least three sets of results: (1) point estimates and confidence intervals for the impact of international laws in each field on any outcome; (2) point estimates and confidence intervals for the impact of international laws from any field on particular outcomes; and (3) a list of the conditions thus far found to potentially mediate the impact of international laws. Meta-analysis will facilitate the first two lines of inquiry, while meta-regression will help answer the third.

#### Meta-analysis

In the earlier scoping review of 90 quantitative impact evaluations [28], most studies were time-series cross-sectional analyses that provided quantitative data on continuous variables suitable for pooling. However, the studies evaluated different international laws across different countries, years, and outcome variables. In this review, we will determine the extent to which it is plausible that effects are similar across the range of the populations, the interventions, and the outcomes; for questions in which it is plausible, we will conduct meta-analyses.

In principle, our aim is to be liberal in this judgment: that is, we are prepared to pool results from a relatively broad range of populations, interventions, and outcomes. Having done that, we can examine the variability in results to determine the extent to which the data support the assumption regarding similar effects across populations, interventions, and outcomes. We anticipate substantial variability and will address this through meta-regression analysis (see below).

Since we know that the studies will measure outcomes of interest in different units, the effect size will be calculated using the difference in mean values divided by the pooled standard deviation of the comparators (e.g., intervention versus non-intervention group or standard deviation before and after introduction of intervention). This calculation will result in a measure of the effect known as the standardized mean difference (SMD) (also sometimes labeled the “effect size”). For each study, the SMD is calculated as the difference in mean outcome between groups divided by the standard deviation of outcome among participants, as represented in the following equation:

$$ \mathrm{S}\mathrm{M}\mathrm{D}=\frac{\mathrm{Difference}\kern0.5em \mathrm{in}\kern0.5em \mathrm{mean}\kern0.5em \mathrm{outcome}\kern0.5em \mathrm{between}\kern0.5em \mathrm{groups}}{\mathrm{Standard}\kern0.5em \mathrm{deviation}\kern0.5em \mathrm{of}\kern0.5em \mathrm{outcome}\kern0.5em \mathrm{among}\kern0.5em \mathrm{participants}} $$

The resulting standard deviations are used to standardize the mean differences to a single scale, as well as in the computation of study weights using the inverse variance method. This method assumes that between-study variation in standard deviations reflects only differences in measurement scales and not differences in the reliability of outcome measures or variability among study populations [35].

The SMD from each study is pooled to provide a summary measure in standard deviation units with an associated confidence interval. Random effects meta-analysis will be used, as this approach is conservative in that it considers both within- and between-study differences in calculating the error term. We will examine the extent of heterogeneity statistically using chi-square tests of heterogeneity and of inconsistency using the *I*^2^ measure.

In interpreting the results, a rule of thumb suggests that 0.2 standard deviation units represents a small effect, 0.5 a moderate effect, and 0.8 a large effect. In addition to this rule of thumb, to facilitate our interpretation, we will convert the SMDs to measures of effect typically used for binary outcomes. In principle, this approach assumes that data are normally distributed, allowing calculation of the probability that results are greater than equal to a particular threshold. These probabilities allow calculation of odds ratios and risk differences. There are a number of methods available to conduct the conversion from continuous to binary outcomes. We will use the approach described by Furukawa [36].

#### Meta-regression analysis

Meta-regression analysis will be used to systematically explore the reasons for different effect sizes and outcomes across studies. Meta-regressions are similar to standard regressions, in which an outcome variable is predicted according to the values of one or more explanatory variables. In meta-regression, the dependent variable is the effect estimate (in this case, the SMD for each study). The independent or explanatory variables are characteristics of studies that might influence the size of the intervention effect. In meta-regression, larger studies have more influence on the relationship than smaller studies, since studies are weighted by the precision of their respective effect estimate. We will use a random-effects meta-regression in which the residual heterogeneity among intervention effects not captured by the independent explanatory variables is incorporated in the same way as in a random effects meta-analysis.

The regression coefficients obtained from a meta-regression analysis will describe how the outcome variable (i.e., the intervention effect) changes with a unit increase in the explanatory variable (i.e., the potential effect modifier). The statistical significance of the regression coefficient is a test of whether there is a linear relationship between intervention effect and the explanatory variable.

The meta-regression will allow us to test existing theories in international law and international relations about when law matters and to provide global policymakers with evidence-informed guidance on when different types of international laws may be most helpful (and when they may even be harmful). Among international lawyers and international relations scholars, the effectiveness of international law stands as a subject of great debate. Elucidating the conditions upon which international law has effects, and what these effects may be, will allow for a strengthened understanding of the way in which international law works.

Specifically, informed by a new analytic framework we have developed [28, 37], we will examine the following a priori specified possible determinants of heterogeneity, including postulated directions of effect. Based on findings from the recent scoping review’s assessment of international laws’ effects [28], we hypothesize larger and more beneficial effects in international laws that deregulate rather than regulate activity, international laws that target countries’ foreign rather than domestic policies, international laws that target low- and middle-income countries rather than high-income countries, international laws assessed in the last decade versus prior decades, and international laws adopted to improve national security or the economy rather than social well-being or the environment [28]. In addition, we will explore the possible impact of study design on magnitude of effect. The study designs included in this regression will include cross-sectional studies, time-series studies of individual jurisdictions, and time-series studies of multiple jurisdictions.

In the absence of direct evidence, the aforementioned analytic framework outlines four criteria intended to assist decision-makers to ensure that new international laws are likely to yield positive effects. It takes into account the uncertain benefits, costs, risks of harm, and trade-offs involved with the implementation of international law. Specifically, the four criteria for assessing international laws are as follows: (1) there must be a significant transnational dimension to the addressed problem; (2) the coercive nature of treaties should be justified by the goals of the proposed solution; (3) there must be a reasonable likelihood that the treaty will achieve benefits; and (4) the treaty should represent the best commitment mechanism among competing alternatives [23]. This systematic review will be used to test these criteria and update the analytic framework.

#### Additional analyses of subgroups

In addition to the meta-regression, we will also do more traditional subgroup analyses. These analyses are currently planned for the following attributes to ascertain effects of different types of international laws under different circumstances:

- *By policy domain*: Analysis across policy domain of international laws such as environment, finance, health, human rights, humanitarianism, and trade will allow for comparison of magnitude of effects of laws operating in different areas of intervention.
- *By enforcement mechanism*: Level or robustness of enforcement mechanisms embedded in treaties or used by states to encourage compliance such as legal penalties, monetary fines, and “naming and shaming” may impact the magnitude of its effects.
- *By impact/indicator measure*: Categorization of studies by impact measures such as economic growth, health status, and trade flows allows for comparison of the effects of international treaties on the same indicators.
- *By scope of the intervention*: This may provide insight into the relationship between the number of countries that are party to an international law and the impact measured on outcomes, such as between an international law binding a small group of countries versus a law adopted through the United Nations which may have almost 200 state parties.
- *By study design*: Robustness of analytical methods may impact the magnitude of effects determined in each study, and categorization by study design will be necessary when we attempt to pool results.
- *By time period*: This may give insight into whether there was enough time to measure an impact and whether international laws adopted at different time periods might have been more successful.
- *By forum*: This may provide information on whether international laws have differing effects depending on the process through which they are created and the organization that may have hosted its negotiation and enforcement, such as the United Nations General Assembly, World Health Organization, and World Trade Organization, or without a formal organization.

## Discussion

This review will use a rigorous systematic methodology and will represent a novel attempt at investigating an important question that has not yet benefitted from systematic synthesis techniques. While the review will build on a recent scoping review [28], it is unlike that previous study in that this planned review: (1) follows a systematic protocol for searching studies, assessing their relevance, extracting information, and summarizing and reporting it; (2) includes more and different types of information (e.g., measure of extent of impact); (3) will use meta-analysis, allowing for the calculation of point estimates and confidence intervals for the effects of past international laws and some factors that influence effects’ direction and depth of impact; and (4) will use meta-regression techniques to systematically draw lessons across international laws, including identifying implementation considerations that influence why some laws may have achieved positive impacts while others did not. By strengthening and extending the methodology of the previous scoping review, this systematic review will be more informative and allow for more precise and unbiased results, thereby facilitating more valuable insights and authoritative conclusions.

This systematic review has several strengths. First, it addresses a particularly salient policy problem, as there exists a widespread and active discussion on the development of new global health laws [38, 39]. These proposed laws have the potential to be beneficial, costly, and/or harmful [37]. This review will help to inform future decisions by identifying what types of international laws produce beneficial effects and under what circumstances. Second, this review follows a systematic and transparent protocol. We will employ recommended and validated methods at all stages of the review, ensuring that the review meets the standards of either, or both, the Cochrane Collaboration’s Public Health Group or the Campbell Collaboration’s International Development Group. Another notable strength is that the scope of the systematic review will be inclusive and comprehensive as we will include gray literature such as dissertations and theses.

An important limitation of the study is its breadth of potential outcomes; the review is very broad, includes many studies, and considers a wide range of factors influenced by international laws. Our application of meta-analysis and meta-regression techniques to these diverse studies and outcomes may be affected by the significant heterogeneity among the pool of eligible studies. This may pose a challenge in comparing the success of different international laws across outcomes as there is much expected heterogeneity between the effects of the laws included. However, our calculation of effect size using SMD will allow us to pool results across different units. Our rigorous approach for measuring heterogeneity will ensure that we only pool results where feasible and appropriate; for example, heterogeneity will be measured prior to meta-analysis. Further, we will continue to have ongoing discussions with statisticians to plan for an efficient and optimal abstraction process in which we collect data that will demonstrate meaningful results. Another important limitation is that while our review includes theses and dissertations, we may have overlooked studies that were not indexed such as government documents or civil society reports.

## Additional files

Additional file 1: PRISMA-P 2015 Checklist. (DOCX 32 kb) Additional file 2: Common Search Strategy Adapted for Ten Electronic Bibliographic Databases and the Dissertations and Theses Database. Adapted search strategy for each searched database. (DOCX 145 kb)

## Abbreviations

CINAHL: Cumulative Index to Nursing and Allied Health Literature
FCTC: Framework Convention on Tobacco Control
GRADE: Grading of Recommendations Assessment Development and Evaluation
PAIS International: Public Affairs Information Service International
PRISMA-P: Preferred Reporting Items for Systematic Reviews and Meta-Analyses
SMD: standard mean difference
TRIPS: Agreement on Trade-Related Aspects of Intellectual Property Rights
