# Supplementary material for: International law’s effects on health and its social determinants: protocol for a systematic review, meta-analysis, and meta-regression analysis
Source: Syst Rev. 2016 Apr 16;5:64. doi: 10.1186/s13643-016-0238-0 (PMC4833910; doi:10.1186/s13643-016-0238-0)
Supplement: Additional file 2: — Common Search Strategy Adapted for Ten Electronic Bibliographic Databases and the Dissertations and Theses Database. Adapted search strategy for each searched database. (DOCX 145 kb) [file 13643_2016_238_MOESM2_ESM.docx]

**Additional file 2: Common Search Strategy Adapted for Ten Electronic Bibliographic Databases and the Dissertations and Theses Database**

**MEDLINE**

Citations yielded = 576

1. (law or laws or agreement* or treaty or treaties or convention* or accord or accords or covenant* or protocol* or charter or charters or regime* or cooperation* or legislation*).tw.

2. (international or global or multi?national or trans?national or foreign or multi?lateral).tw.

3. (quantitative or empirical or experiment or experiments or experimental or quasi-experiment or quasi-experiments or quasi-experimental or statistic* or time?series or cross?sectional or tscs or counterfactual or ANOVA or MANOVA or t-test or z-test or f-test or logistic or correlation or frequentist or Bayesian or maximum likelihood or least squares or parametric or covariance).tw.

4. (effect* or affect* or impact* or ratif* or difference or differences or compliance or comply or adher* or implement* or influenc* or impact* or chang* or measur* or constrain* or screen* or behavio?r or deter* or reduc* or increas* or decreas* or inflat* or vary or variation* or varie*).tw.

5. ((law or laws or agreement* or treaty or treaties or convention* or accord or accords or covenant* or protocol* or charter or charters or regime* or cooperation or legislation*) adj3 (international or global or multi?national or trans?national or foreign or multi?lateral)).tw.

6. 3 and 4 and 5

**Global Health**

Citations yielded = 146

1. (law or laws or agreement* or treaty or treaties or convention* or accord or accords or covenant* or protocol* or charter or charters or regime* or cooperation* or legislation*).tw.

2. (international or global or multi?national or trans?national or foreign or multi?lateral).tw.

3. (quantitative or empirical or experiment or experiments or experimental or quasi-experiment or quasi-experiments or quasi-experimental or statistic* or time?series or cross?sectional or tscs or counterfactual or ANOVA or MANOVA or t-test or z-test or f-test or logistic or correlation or frequentist or Bayesian or maximum likelihood or least squares or parametric or covariance).tw.

4. (effect* or affect* or impact* or ratif* or difference or differences or compliance or comply or adher* or implement* or influenc* or impact* or chang* or measur* or constrain* or screen* or behavio?r or deter* or reduc* or increas* or decreas* or inflat* or vary or variation* or varie*).tw.

5. ((law or laws or agreement* or treaty or treaties or convention* or accord or accords or covenant* or protocol* or charter or charters or regime* or cooperation or legislation*) adj3 (international or global or multi?national or trans?national or foreign or multi?lateral)).tw.

6. 3 and 4 and 5

**CINAHL**

Citations yielded = 391

TX ( (law or laws or agreement* or treaty or treaties or convention* or accord or accords or covenant* or protocol* or charter or charters or regime* or cooperation or legislation*) n3 (international or global or multi?national or trans?national or foreign or multi?lateral) ) AND TX ( quantitative or empirical or experiment or experiments or experimental or quasi-experiment or quasi-experiments or quasi-experimental or statistic* or time?series or cross?sectional or tscs or counterfactual or ANOVA or MANOVA or t-test or z-test or f-test or logistic or correlation or frequentist or Bayesian or maximum likelihood or least squares or parametric or covariance ) AND TX ( effect* or affect* or impact* or ratif* or difference or differences or compliance or comply or adher* or implement* or influenc* or impact* or chang* or measur* or constrain* or screen* or behavio?r or deter* or reduc* or increas* or decreas* or inflat* or vary or variation* or varie* )

**PAIS INTERNATIONAL**

Citations yielded = 831

((all(legislation*) OR all(law) OR all(laws) OR all(agreement*) OR all(treaty) OR all(treaties) OR all(convention*) OR all(accord) OR all(accords) OR all(covenant*) OR all(protocol*) OR all(charter) OR all(charters) OR all(regime*) OR all(cooperation*)) NEAR/3 (all(international) OR all(global) OR all(multi?national) OR all(trans?national) OR all(foreign) OR all(multi?lateral))) AND (all(quantitative) OR all(empirical) OR all(experiment) OR all(experiments) OR all(experimental) OR all(quasi-experiment) OR all(quasi-experiments) OR all(quasi-experimental) OR all(statistic*) OR all(time?series) OR all(cross?sectional) OR all(TSCS) OR all(counterfactual) OR all(ANOVA) OR all(MANOVA) OR all(t-test) OR all(z-test) OR all(f-test) OR all(logistic) OR all(correlation) OR all(frequentist) OR all(Bayesian) OR all("maximum likelihood") OR all("least squares") OR all(parametric) OR all(covariance)) AND (all(effect*) OR all(affect*) OR all(impact*) OR all(ratif*) OR all(difference) OR all(differences) OR all(compliance) OR all(comply) OR all(adher*) OR all(implement*) OR all(influenc*) OR all(impact*) OR all(chang*) OR all(measur*) OR all(constrain*) OR all(screen*) OR all(behavio?r*) OR all(deter*) OR all(reduc*) OR all(increas*) OR all(decreas*) OR all(inflat*) OR all(vary) OR all(variation*) OR all(varie*))

**Worldwide Political Science Abstracts**

Citations yielded = 2353

(all(legislation*) OR all(law) OR all(laws) OR all(agreement*) OR all(treaty) OR all(treaties) OR all(convention*) OR all(accord) OR all(accords) OR all(covenant*) OR all(protocol*) OR all(charter) OR all(charters) OR all(regime*) OR all(cooperation*)) NEAR/3 (all(international) OR all(global) OR all(multi?national) OR all(trans?national) OR all(foreign) OR all(multi?lateral)) AND (all(quantitative) OR all(empirical) OR all(experiment) OR all(experiments) OR all(experimental) OR all(quasi-experiment) OR all(quasi-experiments) OR all(quasi-experimental) OR all(statistic*) OR all(time?series) OR all(cross?sectional) OR all(TSCS) OR all(counterfactual) or all(ANOVA) OR all(MANOVA) OR all(t-test) OR all(z-test) OR all(f-test) OR all(logistic) OR all(correlation) OR all(frequentist) OR all(Bayesian) OR all(“maximum likelihood”) OR all(“least squares”) OR all(parametric) OR all(covariance)) AND (all(effect*) OR all(affect*) OR all(impact*) OR all(ratif*) OR all(difference) OR all(differences) OR all(compliance) OR all(comply) OR all(adher*) OR all(implement*) OR all(influenc*) OR all(impact*) OR all(chang*) OR all(measur*) OR all(constrain*) OR all(screen*) OR all(behavio?r*) OR all(deter*) OR all(reduc*) OR all(increas*) OR all(decreas*) OR all(inflat*) OR all(vary) OR all(variation*) OR all(varie*))

**International Political Science Abstracts**

Citations yielded = 3730

(legislation* OR law OR laws OR agreement* OR treaty OR treaties OR convention* OR accord OR accords OR covenant* OR protocol* OR charter OR charters OR regime* OR cooperation*) AND (international OR global OR multi%3Fnational OR trans%3Fnational OR foreign OR multi%3Flateral) AND (quantitative OR empirical OR experiment OR experiments OR experimental OR quasi-experiment OR quasi-experiments OR quasi-experimental OR statistic* OR time%3Fseries OR cross%3Fsectional OR TSCS OR counterfactual or ANOVA OR MANOVA OR t-test OR z-test OR f-test OR logistic OR correlation OR frequentist OR Bayesian OR “maximum likelihood” OR “least squares” OR parametric OR covariance) AND (effect* OR affect* OR impact* OR ratif* OR difference OR differences OR compliance OR comply OR adher* OR implement* OR influenc* OR impact* OR chang* OR measur* OR constrain* OR screen* OR behavio%3Fr* OR deter* OR reduc* OR increas* OR decreas* OR inflat* OR vary OR variation* OR varie*)

**International Bibliography of the Social Sciences**

Citations yielded = 1788

((all(legislation*) OR all(law) OR all(laws) OR all(agreement*) OR all(treaty) OR all(treaties) OR all(convention*) OR all(accord) OR all(accords) OR all(covenant*) OR all(protocol*) OR all(charter) OR all(charters) OR all(regime*) OR all(cooperation*)) NEAR/3 (all(international) OR all(global) OR all(multi?national) OR all(trans?national) OR all(foreign) OR all(multi?lateral))) AND (all(quantitative) OR all(empirical) OR all(experiment) OR all(experiments) OR all(experimental) OR all(quasi-experiment) OR all(quasi-experiments) OR all(quasi-experimental) OR all(statistic*) OR all(time?series) OR all(cross?sectional) OR all(TSCS) OR all(counterfactual) OR all(ANOVA) OR all(MANOVA) OR all(t-test) OR all(z-test) OR all(f-test) OR all(logistic) OR all(correlation) OR all(frequentist) OR all(Bayesian) OR all("maximum likelihood") OR all("least squares") OR all(parametric) OR all(covariance)) AND (all(effect*) OR all(affect*) OR all(impact*) OR all(ratif*) OR all(difference) OR all(differences) OR all(compliance) OR all(comply) OR all(adher*) OR all(implement*) OR all(influenc*) OR all(impact*) OR all(chang*) OR all(measur*) OR all(constrain*) OR all(screen*) OR all(behavio?r*) OR all(deter*) OR all(reduc*) OR all(increas*) OR all(decreas*) OR all(inflat*) OR all(vary) OR all(variation*) OR all(varie*))

**Social Sciences Abstracts**

Citations yielded = 602

TX ( ((legislation* OR law OR laws OR agreement* OR treaty OR treaties OR convention* OR accord OR accords OR covenant* OR protocol* OR charter OR charters OR regime* OR cooperation*) N3 (international OR global OR multinational OR transnational OR foreign OR multilateral)) ) AND TX ( (quantitative OR empirical OR experiment OR experiments OR experimental OR quasi-experiment OR quasi-experiments OR quasi-experimental OR statistic* OR time?series OR cross?sectional OR TSCS OR counterfactual or ANOVA OR MANOVA OR t-test OR z-test OR f-test OR logistic OR correlation OR frequentist OR Bayesian OR “maximum likelihood” OR “least squares” OR parametric OR covariance) ) AND TX ( (effect* OR affect* OR impact* OR ratif* OR difference OR differences OR compliance OR comply OR adher* OR implement* OR influence* OR impact* OR chang* OR measur* OR constrain* OR screen* OR behavio?r* OR deter* OR reduc* OR increas* OR decreas* OR inflat* OR vary OR variation* OR varie*) )

**Applied Social Sciences Index and Abstracts**

Citations yielded = 240

((all(legislation*) OR all(law) OR all(laws) OR all(agreement*) OR all(treaty) OR all(treaties) OR all(convention*) OR all(accord) OR all(accords) OR all(covenant*) OR all(protocol*) OR all(charter) OR all(charters) OR all(regime*) OR all(cooperation*)) NEAR/3 (all(international) OR all(global) OR all(multi?national) OR all(trans?national) OR all(foreign) OR all(multi?lateral))) AND (all(quantitative) OR all(empirical) OR all(experiment) OR all(experiments) OR all(experimental) OR all(quasi-experiment) OR all(quasi-experiments) OR all(quasi-experimental) OR all(statistic*) OR all(time?series) OR all(cross?sectional) OR all(TSCS) OR all(counterfactual) OR all(ANOVA) OR all(MANOVA) OR all(t-test) OR all(z-test) OR all(f-test) OR all(logistic) OR all(correlation) OR all(frequentist) OR all(Bayesian) OR all("maximum likelihood") OR all("least squares") OR all(parametric) OR all(covariance)) AND (all(effect*) OR all(affect*) OR all(impact*) OR all(ratif*) OR all(difference) OR all(differences) OR all(compliance) OR all(comply) OR all(adher*) OR all(implement*) OR all(influenc*) OR all(impact*) OR all(chang*) OR all(measur*) OR all(constrain*) OR all(screen*) OR all(behavio?r*) OR all(deter*) OR all(reduc*) OR all(increas*) OR all(decreas*) OR all(inflat*) OR all(vary) OR all(variation*) OR all(varie*))

**Social Sciences Citation Index**

Citations yielded = 6887

TOPIC:((legislation* OR law OR laws OR agreement* OR treaty OR treaties OR convention* OR accord OR accords OR covenant* OR protocol* OR charter OR charters OR regime* OR cooperation*) SAME (international OR global OR multi?national OR trans?national OR foreign OR multi?lateral))*AND* TOPIC: ((quantitative OR empirical OR experiment OR experiments OR experimental OR quasi-experiment OR quasi-experiments OR quasi-experimental OR statistic* OR time?series OR cross?sectional OR TSCS OR counterfactual or ANOVA OR MANOVA OR t-test OR z-test OR f-test OR logistic OR correlation OR frequentist OR Bayesian OR maximum likelihood OR least squares OR parametric OR covariance)) *AND* TOPIC: ((effect* OR affect* OR impact* OR ratif* OR difference OR differences OR compliance OR comply OR adher* OR implement* OR influenc* OR impact* OR chang* OR measur* OR constrain* OR screen* OR behavio?r* OR deter* OR reduc* OR increas* OR decreas* OR inflat* OR vary OR variation* OR varie*))

**Dissertations & Theses**

Citations yielded = 3075

(all(legislation*) OR all(law) OR all(laws) OR all(agreement*) OR all(treaty) OR all(treaties) OR all(convention*) OR all(accord) OR all(accords) OR all(covenant*) OR all(protocol*) OR all(charter) OR all(charters) OR all(regime*) OR all(cooperation*)) NEAR/3 (all(international) OR all(global) OR all(multi?national) OR all(trans?national) OR all(foreign) OR all(multi?lateral)) AND (all(quantitative) OR all(empirical) OR all(experiment) OR all(experiments) OR all(experimental) OR all(quasi-experiment) OR all(quasi-experiments) OR all(quasi-experimental) OR all(statistic*) OR all(time?series) OR all(cross?sectional) OR all(TSCS) OR all(counterfactual) or all(ANOVA) OR all(MANOVA) OR all(t-test) OR all(z-test) OR all(f-test) OR all(logistic) OR all(correlation) OR all(frequentist) OR all(Bayesian) OR all(“maximum likelihood”) OR all(“least squares”) OR all(parametric) OR all(covariance)) AND (all(effect*) OR all(affect*) OR all(impact*) OR all(ratif*) OR all(difference) OR all(differences) OR all(compliance) OR all(comply) OR all(adher*) OR all(implement*) OR all(influenc*) OR all(impact*) OR all(chang*) OR all(measur*) OR all(constrain*) OR all(screen*) OR all(behavio?r*) OR all(deter*) OR all(reduc*) OR all(increas*) OR all(decreas*) OR all(inflat*) OR all(vary) OR all(variation*) OR all(varie*))
